# Dysmenorrhœa

**Published:** 1910-05-07

**Authors:** R. A. Gibbons

**Affiliations:** Physician to the Grosvenor Hospital for Women.


### Hospital Clinics.

### DYSMENORRHŒA.

By R. A. GIBBONS, M.D., F.R.C.S.; Physician to the Grosvenor Hospital for Women.

(A Lecture delivered at the Medical Graduates' College and Polyclinic, January 26, 1910.)

Gentlemen,—The subject we have to discuss is one which ought to be of interest to every practitioner of medicine, for this affection is apt to be the means of suffering from the time a girl is about 12 or 14 during the many years up to the menopause; although the worst cases come under the observation of the gynæcologist, yet before doing so they pass through the hands of the family physician, so that there is really none of the ordinary diseases of women for which advice is more frequently sought. It so frequently shatters a girl's health before she has become a woman that too much attention cannot be paid to the careful study of this disease. I am certain that the judicious management of dysmenorrhœa prevents, in many cases, girls and women drifting into chronic invalidism. Although many regard dysmenorrhœa as a symptom of something occurring internally, it is certainly a disease. Healthy girls never have pain with menstruation; but unfortunately only the minority of women menstruate in this manner, suffering no pain. It has been estimated that only about one-third of all women menstruate without pain.

I propose to deal with this subject principally under the head of ætiology, before passing on to what is, of course, the most important consideration for us, namely, treatment. In the first place, I shall merely mention the divisions into which dysmenorrhœa has been classified by others. It is usually said that the forms of dysmenorrhœa are idiopathic, congestive, membranous, spasmodic, obstructive, ovarian, and even neurasthenic. Some believe there is a form of dysmenorrhœa which is purely nervous, and which may be due to faulty innervation of the uterus, be it of an anatomic or a functional defect. This variety they speak of as neuralgic dysmenorrhœa.[1]

### Varieties of Dysmenorrhœa.

Others think that it is easy to divide the various forms clinically into uterine[2] and extra-uterine, and others, again, have classified them into primary and acquired. The greater experience one has, the more convinced one is that these divisions are entirely unnecessary. I know of nothing to make me alter the view I have long held, that there is only one form of dysmenorrhœa, *i.e.* spasmodic, and that, therefore, all other varieties or so-called varieties are merely confusing. If the word dysmenorrhœa were confined to the disease to which, in my opinion, it ought only to be applied, that is, painful contractions of the uterus during menstruation, confusion could not arise; but because it is so frequently associated with affections of the ovaries, Fallopian tubes, or certain conditions of the nervous system, many forms of the disease have been described. To speak of neurasthenic dysmenorrhœa means only that a woman suffering from neurasthenia has dysmenorrhœa superadded to her condition, and in such a case it is quite possible that the one attending her or prescribing for her may think that dysmenorrhœa has supervened in the neurasthenic woman, and that therefore neurasthenic dysmenorrhœa is a legitimate name for the affection. But as a matter of fact pain is relative, and anything which lowers the tone of the nervous system, such as the onset of menstruation, is apt to bring on pain in certain parts, or to aggravate the sensation from very slight to severe. The most important division of dysmenorrhœa and one which demands a considerable amount of attention, owing to the fact that it is always so constantly referred to, is obstructive, or membranous dysmenorrhœa. It is considered that membranous dysmenorrhœa produces obstruction on account of the membranes or shreds which are said to block the cervical canal, thus preventing a free flow, and causing spasm of the uterus. As I have already said, I do not acknowledge that there is any real obstructive form. It will be found on examination that many women pass membranes or shreds, which are only known about when inquired into. Ante-flexion is also said to produce obstruction, but it is well known that obstruction is not absolutely present. According to a paper by Dr. Gustav Kolischer,[3] who has observed certain cases, the blood normally flows in a steady stream from the external orifice to the posterior fornix of the vagina only occasionally, and then gradually increasing and decreasing in volume, but in painful menstruation during attacks the blood is forced out in jets from the external os, evidently by forcible contractions of the uterine body.

All those who have constantly examined cases before operating for dysmenorrhœa know by experience that although the ordinary uterine sound cannot always be admitted, yet a probe of smaller size can invariably be passed into the cavity of the uterus, showing that there is no actual obstruction in the cervical canal. The late Dr. Matthews Duncan used only to speak of one form of dysmenorrhœa, although he mentions two in his lectures, and Dr. Herman [4] also considers that there is only one form—dysmenorrhœa spasmodica.

### Symptoms of Dysmenorrhœa.

It may be here convenient to speak of the ordinary symptoms of dysmenorrhœa. In many cases those women who suffer from it have premonitory symptoms for some days of malaise—slight pain or discomfort in the back, shooting pains, slight or severe, down the thighs, lassitude, disinclination for exertion, abdominal pain, nausea, and headache. These symptoms may last from a few hours to several days, but are only the precursors of the regular pains, which occur either before or during the onset, or after the flow has been thoroughly established. The contractions come in waves or paroxysms, and in the most severe form the contractions may affect the bladder and rectum, producing strangury and tenesmuus, as well as violent abdominal bearing-down pain. These pains or violent contractions are the same in character as labour pains, and last from one to two or three minutes. There is no regularity, except, as a rule, in the individual herself, of these symptoms. Each case must be judged by itself, for although most of them can be grouped into those commencing in a certain manner, none are exactly alike. I have divided the cases coming under my observation into slight, severe, and agonising. In the divisions I have made, those coming under the head of "agonising" are not common. The word ought only to be applied to a woman evidently in the most excruciating pain, who cannot keep still for an instant, but rolls about the bed, is covered with perspiration, becomes cold, and, finally, faints from the pain. Of such I have seen a fair number, and only recently, in a case where I operated, the patient became collapsed each month from excessive pain. In another case, brought to me some years ago, a lady about twenty-four years of age developed maniacal excitement every month, had to be provided with two nurses, and invariably became collapsed. In her case complete relief was obtained by dilatation, and she never had any trouble afterwards. Sometimes the usual monthly attack comes on quite suddenly. It is very common for a girl to be awakened by abdominal pain, which increases in severity until it reaches the height to which she is accustomed, when it remains the usual time, and then gradually disappears. When the pain increases in severity, nausea is induced, and very often vomiting follows, in some cases frequent, in some seldom. I have known persistent vomiting brought on regularly by dysmenorrhœa, lasting for some hours or days, and leaving the patient weak and ill, so that a large number of days pass before regaining strength, which is again to be reduced by the next attack. In the most severe form I have never known pain to last more than a few hours, but there may be considerable clonic spasms for twenty-four hours or more. A fair estimate may be gained of the severity of the attack by asking the patient if she can fulfil her social or other duties during the time of menstruation. A patient will often talk of "agony of pain," and on further questioning one can ascertain that she will be able to attend a ball or fulfil some engagement which gives pleasure, when it may certainly be said that she does not suffer severely. I invariably ascertain if a patient can keep her engagements during the period of pain. If she cannot it may be fairly assumed that she suffers. Sometimes there is a sensitive condition of the skin from reflected pain in the region supplied by the sensory nerves from the eleventh dorsal to the second lumbar segments of the spinal cord.

### The Capacity for Bearing Pain.

It may here be well to say a few words about the extraordinary difference there is among girls and women of all classes in bearing pain. Some girls, accustomed to every care and luxury, retire to bed every month, saying they have great pain, when, if they were carefully watched, it would be evident that they were not suffering severely. On the other hand, I have known women who have been obliged to make their living keep at their work unless prevented by actual sickness or faintness from doing so, notwithstanding intense suffering. The test I have for examination in hospital is whether a patient can remain at work during the time of the period. If she cannot, it means sick leave, and after a short time loss of situation, so that she is driven to the hospital for radical treatment. Doubtless many women, suffering severely, will continue to do their regular work without interruption; but if actual vomiting or faintness be brought on they are obliged to desist. Some divide the symptoms into "uterine," where there is pain in the back with other symptoms supposed to have special reference to the uterus, and "ovarian," where the pain is particularly referred to one or both ovarian regions. It is undoubted that many women complain much more of pain in one or the other side of the abdomen, or in the hypogastric region only, and that these may not suffer particularly from pain in the back or down the thighs; but I do not regard any refinement of symptoms as necessary. Generally speaking, the pain is pelvic, and although it would be easy to tabulate a certain number as suffering from sacral ache, and others as complaining mostly of aching and pain down the thighs, I do not consider that any useful object is to be attained.

I do not think I need take much time in discussing the diagnosis of the disease. It is so common, and the patient herself is so accustomed usually to the recurring condition, that a mistake can hardly be made. Nevertheless, it is well to bear in mind that other conditions may occur at the same time, and possibly be overlooked. Although there may be in some cases of dysmenorrhœa extreme tenderness all over the abdomen, it is hardly likely to be confounded with peritonitis. Absence of temperature, characteristic pulse, and the usual symptoms associated with peritonitis are sufficient for the diagnosis. The cases where the friends or the patients themselves may be alarmed and in doubt are those in which, although severe dysmenorrhœa is regular, yet for some reason the attack may be intensified, and medical advice sought. Under such circumstances I once saw a patient who was suffering from strangulated hernia, and who was very ill when I saw her. She was operated on successfully. It is well also to bear in mind that in cases of peritonitis absolute rest in the recumbent position is the most grateful to the patient, whereas in severe dysmenorrhœa she will not keep still for an instant. In the case of membranous dysmenorrhœa it is not always possible, without a microscopic examination, to say whether the cast is menstrual or not. There are vaginal casts, and decidual, as well as casts of the bladder from inflammation, the former being thrown off in exfoliative vaginitis, and if this be accompanied by severe pain it might be easily mistaken for membranous dysmenorrhœa. Of course, it is possible also for the two latter affections to occur together.[5]

And now as to the ætiology of this affection. I look upon the cause of the pain of dysmenorrhœa as a spasm or cramp of the uterus. Most people know what severe pain may be produced by cramp of the muscles of the calf. The uterine spasm may be tonic or clonic, and I have a theory that when in the severe cases there is a true tonic contraction, it is this which brings about the actual collapse from the severity of the pain. The clonic contractions may continue indefinitely, causing severe pain, but pain which comes and goes, and which does not produce the profound impression on the nervous system caused by the tonic contraction. It is important to remember that the painful contractions occur at menstruation, for although ovulation and menstruation come about the same time, ovulation often follows menstruation, and may occur between menstrual periods. The ovarian changes which precede ovulation, by producing ovarian tension, reflexly excite the uterus and cause menstruation, but may set up much pain before its arrival, which I shall refer to later on.[6]

### A Note on Causation.

Now, what we have to ask is: What can be the cause of these painful uterine contractions? Dysmenorrhœa is a disease, and must have a cause for its production. Many are ready to give various explanations; I confess I know nothing at the present moment which will be accepted as the true explanation of this painful contraction. The most common explanation is the "obstructive dysmenorrhœa," or mechanical theory, to which I have already referred; and if the textbooks are consulted they will be found to give this theory, and to explain it by saying that there is ante-flexion, which obstructs menstrual flow. It has been suggested that, as the result of ante-flexion and consequent obstruction, a little blood is forced through the Fallopian tubes every month into the peritoneal cavity, giving rise to miniature hæmatocele. These small hæmorrhages are considered by some to be the cause of the posterior perimetritis which sometimes accompanies ante-flexion.[7] As I have already said, I do not accept this theory of obstruction, for I do not believe, however acute the ante-flexion may be, that the flow is hindered sufficiently to influence the uterus in producing pain. The most striking argument to be adduced in support of this is hæmatocolpos, where we know that there is a complete blocking of the exit from the uterus, and yet no pain, in some cases, is complained of. In addition to this, we know that more than half the cases of dysmenorrhœa come on in women who have not borne children, after the period has been regularly and painlessly established. Again, I have operated in some cases of sterility when only the finest probe could be passed through a pin-hole os, and yet no pain has been complained of at menstruation, and there was the usual normal amount. No amount of acute ante-flexion, or retro-flexion blocks the canal sufficiently to prevent gradual flow, however slight it may be, from the cervix, and therefore this really cannot be considered a sufficient explanation of the pain at the period. The average amount of blood lost during menstruation is about 4 oz., and if this is distributed over, say, four days, it gives an average loss of one-third of a drop in every minute, which will not be difficult to pass along any cervical canal.

### General and Local Causes.

Now, it is quite easy to make a large number of headings under "general" and "local" causes, uterine or extra-uterine, primary or acquired; but this serves no practical purpose, for we are no further forward in arriving at an explanation. It is usual to advance chronic metritis or pelvic congestions as the cause; but if we consider this for a few moments we shall see that it is no explanation at all. Women suffering from metritis or endometritis are liable to have pain or discomfort increased at the time of the period, for the whole uterus and adnexa become more charged with blood, and the tension is raised. It is, therefore, natural that discomfort and pain should be increased, for we know how much tension in the vessels has to do with pain. In all women, as menstruation comes on, there is a rise in vascular tension, as well as a slight rise in temperature, and because in dysmenorrhœa it may be proved that metritis or endometritis, or acute ante-flexion or retro-flexion is present, the conclusion is arrived at by some that it is the cause of the pain. The menstruation only aggravates the pain which has been produced by the congestion present, with the origin of which menstruation has nothing to do. This applies to all pelvic affections, whether of the uterus, tubes, or ovaries. But this source of origin, even if it did exist in married women, could not be adduced as a cause in virgins, for I know of no work on pathology which states that endometritis is common in them, whereas we know that dysmenorrhœa is. If it occurs at all, it must be rarely. In membranous dysmenorrhœa it might be that there was probably some obstruction, and that the spasmodic pain was caused by the efforts of the uterus to expel the membrane. But the expulsion of the membranes or complete casts of the inner surface of the uterus is frequently associated with dysmenorrhœa, as is familiar to all, and, indeed, it is often the presence of these membranes or complete casts which induces a patient to seek medical advice. That they are not the cause of the pain is proved by the knowledge that these casts may be expelled without any pain at all.

If certain questions are put with reference to menstruation, it will frequently be found that shreds or membranes are passed without the least pain quite commonly. I have had cases which have come before me only because a complete cast of the inner surface of the uterus has been passed on one occasion, and although the patient has known that she has regularly passed shreds, she has paid no attention to them until alarmed by the presence of the cast. In this membranous dysmenorrhœa much depends upon the period of degeneration in the menstruation, which I need not go into now; but it may be shortly said that the mucous membrane of the uterus exhibits—(1) the period of growth in the five days preceding menstruation, characterised by a rapid increase of the stroma, blood-vessels, epithelium, etc., of the membrane; (2) four days of menstruation, or period of degeneration, during which the capillary hæmorrhage takes place, and the epithelium suffers degenerative changes, and is cast off more or less; (3) the seven days period of regeneration, during which the mucous membrane returns to its normal state; (4) the twelve days period of rest, during which the endometrium remains in a quiescent state.[8] Gellheim explains this pain as being caused by dragging on the peritoneum,[9] an explanation which will not suffice in a normal pelvis, but may serve when the mechanism of the dragging process is considered in those cases where direct or indirect adhesions between the uterus and parietal serosa are found.

According to Dr. Mathes,[10] there is a causal connection between dysmenorrhœa and enteroptosis. Kermanner says that it is nearer the truth, and analogous to the opinions held by Lennander, to seek the source of dysmenorrhœic pain not in the uterus but in the pelvic tissues, and in those divisions of it rich in nerves, the sacro-uterine ligament for example. Tobler [11] remarks that as regards the significance of menstrual pain in nullipari: "It is important to ascertain whether it has existed from puberty (primary dysmenorrhœa), or whether it has developed later (secondary dysmenorrhœa), the latter being more frequent—as much as 58 per cent. Both primary as well as secondary dysmenorrhœa are found with extraordinary frequency in girls with constitutional affections (chlorosis). He considers it is probable that the most important seat of origin of the pain is just where the most extensive vascular and nervous plexuses are present, namely, in the sub-peritoneal connective tissue. In this way, the kind of dysmenorrhœic pain, certainly that which is most present in nullipari, may be attributed to stretching and tearing of the sub-peritoneal nerves, as has lately been maintained in the case of other pains in the abdominal cavity.

### Some Theories.

It must be borne in mind that all uterine contractions are not painful. It is now known that there are contractions continually going on in the unimpregnated uterus, and these are intensified at menstruation. They are sufficient to get rid of blood, degenerated mucous membrane, or complete casts of the uterine cavity, without causing pain to the individual; but the contractions with which we have now to deal must be considered to be morbid. In some, these contractions are intensified by a small or large fibroid in the wall of the womb, or by a polypus in the cavity setting up irritation, and acting as a foreign body. It is most important to remember that if a woman who has menstruated regularly and naturally for many years seeks advice because of increasing dysmenorrhœa, it is extremely suspicious, and at once a fibroid or a polypus must be thought of as the cause. This is one of those affections classed under "obstructive dysmenorrhœa"; but, as already pointed out, in my view, it ought not to be spoken of as such. All who are in the habit of seeing many cases of dysmenorrhœa have, doubtless, some theory to account for these morbid contractions. Herman (*op. cit.*), whose writings are always admirably clear, says that his theory is "that dysmenorrhœa exists because the centres in the spinal cord or in the sympathetic system which regulate the movements of the genital canal are imperfectly developed." In the normal painless menstruation there are contractions of the body of the uterus and dilatation of the cervix, so that the menstrual flow is expelled without pain or difficulty. His theory is that in dysmenorrhœa this natural dilatation of the cervix is absent, and in consequence the contractions of the uterine body are morbidly violent and painful. I agree with Herman in thinking that the pain of dysmenorrhœa is caused by painful contractions of the body of the uterus, and that the dilatation of the cervix which normally ought to take place does not occur; but I do not think that his theory can be altogether accepted, although it is nearly right, for otherwise I do not believe that the disease could be cured by dilatation, or the relief would be so temporary that constant dilatation would have to be undertaken because of the undeveloped nerve centre. But the strongest argument against it is that dysmenorrhœa may be induced or occur long after the flow has been regularly and painlessly established—what has been called by some secondary dysmenorrhœa, in contradistinction to primary dysmenorrhœa, beginning with the onset of menstruation, and about this I may speak later; but I may here say that if the spinal centre were imperfectly developed, then the onset of the flow must be painful, and continue to be so; but we know that this is not always the case, and the most severe cases of dysmenorrhœa may have remissions. If the imperfectly developed centres became gradually developed, and the dysmenorrhœa ceased, then it would be difficult to explain why there should be a recurrence of the pain. My own theory of this disease is that it is caused by the circulation in the blood of some bio-chemical material, acting on the hyperæsthetic mucous membrane of the uterus, and possibly also upon the nerve centres regulating the genital canal, and that this material is due to faulty secretions of the ovaries. My ground for this is that if any ordinary healthy woman is examined with a uterine sound, directly it passes into the region of the internal os and beyond she complains of discomfort, or, at any rate, it is disagreeable to have the mucous membrane, which is normally very sensitive, touched, but she can bear the examination perfectly well. Now, if you examine a woman who suffers from dysmenorrhœa with a probe, directly it passes into the internal os she will say that it is extremely painful, and if you continue to introduce the probe so that it touches the uterine mucous membrane, the patient may declare that it is intolerable. Now afterwards, on withdrawal of the sound, she will say that the pain she complains of is exactly the same as at the time of the period, and this same kind of pain may continue for some time after the examination. The touching of the mucous membrane by the sound produces apparently a reflex action, and a painful contraction of the uterus ensues. Anything which acts as a foreign body will do the same, and this, in my opinion, is the explanation of the painful contraction in membranous dysmenorrhœa: the shreds or casts irritate the hyperæsthetic mucous membrane. It seems to me that as the onset of menstruation occurs, and an increased secretion commences, which is sufficient in a condition of hyperæsthesia to irritate the mucous membrane in such a manner that reflex painful contractions occur, these spasms continue until the mucous membrane becomes adapted to the continued irritation, or, in other words, because the irritability of the uterus is exhausted, and pain no longer occurs, or the source of irritation in the circulation is no longer present when the reflex action ceases. As in the study of this disease there is this irritable mucous membrane, which is the only part we know must be morbidly affected, it seems reasonable to assume that in some way it accounts for the spasm of the uterus. It is not, however, easy to explain what causes these changes of the mucous membrane to bring about this hyperæsthesia—whether the irritation is due to some secretion or altered blood it is impossible to say. We know from Gautier and Douser[12] that menstrual discharge contains four times as much iodine as normal blood, and an amount of arsenic equal to the whole quantity normally present in the thyroid gland.[13] It is, therefore, possible that this is sufficiently irritating to act on the hyperæsthetic mucous membrane. But in any case, there evidently seems to be an irritant which, acting on a morbidly hyperæsthetic mucous membrane, or directly on the nerve centres, produces painful contractions of the womb. Matthews Duncan likened this disease to spasmodic asthma, where there is also doubtless a hyperæsthetic mucous membrane of the bronchial tubes, difficult of explanation, and what we have to account for is the morbidly hyperæsthetic condition of the mucous membrane of the uterus. It is well known that at the onset of the period there is an alteration of the blood-pressure. After rising, it falls slowly, the maximum of fall being coincident with the appearance of the flow.[14] This may be due to the action of the ovarian secretion influencing the spinal centre for the genital canal or region, and vaso-motor areas. It is possible that this ovarian secretion, which undoubtedly has a powerful influence on the blood, may, under certain conditions of health, contain an irritating material, which acts on the mucous membrane of the uterus. When the amount or properties of the ovarian juice or secretion is absolutely normal, no alteration takes place in the regular menstruation, but when it contains some abnormal constituent, or its elements are not duly proportioned, then the action on the blood causes the secretion preceding menstruation to irritate the uterine mucous membrane, and give rise to the reflex action leading to painful contraction. It is probable that in time we shall know more of the functions of this ovarian secretion, but at present we can only theorise as to its possibilities. It certainly seems reasonable to believe that the proper action of the ovaries and their secretion must have everything to do with menstruation being carried on in a perfectly normal manner. We now know that upon the equilibrium of the blood glands depends our health, and as our knowledge becomes extended we may hope to ascertain exactly the action of the ovarian secretion.

Fellner[15] says that the uterus secretes internally, that this secretion forms a part of those toxins which affect the increase in the wave, so that the ovary becomes poisoned, and that between secretion and organs a relationship of dependence exists. That ovarian secretion is discharged is supported by the fact that calcium is secreted by the uterus,[16] and on the other hand, that the functional activity of the ovary proceeds concurrently with the excretion of calcium.

### Ovarian Dysmenorrhœa.

Riebold[17] considers that concurrently with the strictly periodical fulfilment of the process of ovulation certain secretions obtain access to the circulation. These, under physiological conditions, cause, first an increase in nervous and psychic irritation, and, secondly, an increase in the blood-pressure and vital energy, the general heightening of the process of metabolism, and consequently the more generally severe hyperæmisation of the internal organs, and a more active secretion of fluids. These processes may be set up independently of uterine hæmorrhage. He mentions what he calls "recurrent rheumatoid ovulation fever" occurring in young girls, accompanied by more or less severe disturbance of the general condition, at the time of menstruation. He considers that ovulation has the effect of storing up an infective focus of rheumatic material, present in the body but latent, or else that a functional defect of the secretory activity of the ovaries may underlie a diseased condition.

There is another explanation of what is termed by some "Ovarian dysmenorrhœa," when all the pain seems to be limited to one or the other ovarian region or to both, and that is there is sometimes a thickened capsule of the ovary found, with small cysts in the ovary itself, or there may be a tuberculous ovary. It seems that as the increased congestion takes place during menstruation, the ovary swells, the capsule becomes tightened, and great neuralgic pain is set up owing to tension and compression of nerves. This pathological condition is certainly found in some cases of dysmenorrhœa, but is not the cause of the real painful contractions, the spasmodic dysmenorrhœa of which I speak. I believe, when this pathological condition exists in the ovary, there is often severe neuralgic pain, lasting many hours, but it is not of the same character which we have been discussing as spasmodic dysmenorrhœa, although, of course, it may be associated with it. With spasm or intense contraction, all the muscular fibres squeeze the nerves supplying the uterus, and so cause pain. In some cases it is possible that actual neuritis of some of the nerves may take place, leading to continued discomfort in the region of the womb long after the period is over; and Lawson Tait believed that there are nerves from the tubes to the sympathetic system, and these he called "menstruating nerves." It is probable that the continued contraction of the uterus causes a slight amount of hypertrophy of muscular tissue; at any rate, the cavity tends to become elongated. In nearly all cases of well-marked dysmenorrhœa on which I have operated the sound measures an increase of one-quarter to half an inch. This enlargement is more likely to be due to repeated severe contractions of the uterine muscular tissue than to anything else, and is analogous to the ordinary hypertrophy of muscular tissue from overuse.

I do not believe that a small uterus—the so-called infantile uterus—has anything to do with dysmenorrhœa. No doubt it is frequently associated with dysmenorrhœa, but I believe that the cause of dysmenorrhœa is exactly the same as in patients with a normally developed uterus, and the actual ill-developed uterus follows the same laws in this disease as the well-developed one.

As I have so frequently found hypertrophy associated with this disease, I cannot agree with Theilhaber's theory that the pain is due to a badly developed uterine muscle.

(*To be concluded.*)

Notes.

[1] Kelly and Noble. *Gynæcology and Abdominal Surgery,* 1908, p. 235, vol. 1.

[2] Sir Halliday Croom. *Allbutt's System of Gynæcology,* p. 95.

[3] *American Journal of Obstetrics,* 1909, p. 644.

[4] An Address on Dysmenorrhœa, *British Medical Journal, April* 17, 1909.

[5] Kelly. *Med. Gynæc.* 117.

[6] Edgar. *Practice of Obstetrics,* p. 25, 1906.

[7] Sir Halliday Croom. *Allbutt's System of Gynæcology,* p. 97.

[8] Howell. *Text Book of Physiology,* 1908.

[9] Die Erklaerung der Dysmenorrhœa durch Bauchfellzerrung. *Monatsschrift für Geburtshilfe und Gynäkologie,* 1908, XXVII.

[10] Ueber Aetiologie und Therapie der Dysmenorrhœa. *Monatsschrift für Geburtshilfe und Gynäkologie,* 1908, XXVII. pp. 73-77.

[11] Ueber Primaere und sekundaere Dysmenorrhœa. *Monatsschrift für Geburtshilfe und Gynäkologie,* Berlin, 1907, XXVI. p. 801-822.

[12] *Académie de Médecine.*

[13] Leonard Williams. *Clinical Journal,* March 1909.

[14] Mosher. *Johns Hopkins Hospital Bulletin.*

[15] Die wechselseitigen Beziehungen der innersektorischen Organe, insbesondere zum Ovarium. Zugleich ein Beitrag zur Lehre von der Menstruation. *Volkmann's Sammlung Klin. Vorträge* N.F. 508 Interchangeable relations of the normal secretory organs, especially of the ovary. Contribution to the doctrine of menstruation.

[16] Bell. *British Medical Journal,* 1907, vol. 1, p. 920.

[17] Ueber die Wechselbeziehungen zwischen dem Ovulations-Vorgang in der Menstruation und internen Krankheiten. *Münchener Med. Woch.* 1907, XLVII., pp. 1868, 1935.

